# "Am I the only one who will Spread the Virus?": Impact of Public Stigma Towards the East Asian Population Living in Spain Related to COVID-19

**DOI:** 10.1007/s40615-024-02281-w

**Published:** 2025-01-30

**Authors:** Iris María Muñoz-del-Pino, Francisco Javier Saavedra-Macías, Elvira Pérez-Vallejos

**Affiliations:** 1 Department of Experimental Psychology, University of Seville. Faculty of Psychology, Camilo José Cela S/N, 41018 Seville, Spain; 2 Horizon Digital Economy Research, University of Nottingham, School of Medicine, Nottingham, UK

**Keywords:** COVID-19, East Asia, Public stigma, Quality of life, Spain

## Abstract

Previous studies have suggested that COVID-19 led to an increase in stigma towards the Asian population with a negative impact on their health. This study aims to explore this phenomenon and its impact on health through the qualitative analysis of semi-structured interviews conducted with 26 people of Asian origin living in Spain from September 2020 to September 2021. Among the results, it was found that, prior to the pandemic, discrimination was mostly verbal. After the outbreak of the pandemic, some participants, who were blamed and referred to as "COVID", experienced fear and physical aggression. Among the health effects, mental and social disturbances such as feeling like "permanent foreigners", worrying about being stigmatized or fear of interacting with others were prominent. The main protective factor was the support network, including education and community cohesion as main determinants. Future research is needed to analyse the evolution of this stigma after the pandemic and to explore in detail its impact on health**.**

## Introduction

On 30 January 2020, the World Health Organisation (WHO) designated a Public Health Emergency of International Concern (PHEIC) in response to the emergence of a novel coronavirus (SARS-CoV-2, COVID-19) that had rapidly disseminated across global populations, leading to its categorisation as a pandemic on 11 March 2020 [1]. COVID-19 thus became a public health challenge, not only because of its impact on population health care, but also because of its economic and social effects [2].

One of the social consequences of the pandemic was the stigma associated to certain vulnerable groups. It is necessary to distinguish between public stigma and self-stigma. The former is characterised by the social perception that an individual is socially unacceptable, based on one's own characteristics (physical, psychological…) while leading to negative reactions and, consequently, devaluation. The second arises when the stigmatised individual accepts the negative stereotypes associated with him/herself and internalises them [3, 4]. In this paper, public stigma will be studied. This is composed of stereotypes (general negative beliefs about the group, e.g. "Asian people are dangerous because they can spread COVID-19"), and prejudice (emotional reactions associated with the stereotypes) that can lead to discrimination (verbal aggression, physical aggression, social distance, etc.) [3, 5, 6]. Within the concept of public stigma, there is a variant mentioned by Pescosolido & Martin [5], where the stigma associated with group identity, which is physically visible, has merged with disease stigma, whose features are invisible or hidden. There is evidence throughout history such as Haitian people and HIV or African-American men and schizophrenia [5]. According to some authors [6, 7] during the pandemic this identification of a disease with a group occurred with Asians and COVID-19.

Regarding risk factors, the misuse of the media has been referred to as a predictor of public stigma. It is not only the verification of information obtained from these sources that is important, but also the vocabulary and ideologies behind the language. Denominations such as "Chinese virus" or "the virus of the Chinese" have been extracted from speeches of political leaders in the affected countries, giving a politicised perspective of the pandemic (Geisser, 2020; Haokip, 2021). Supporting this cause, Cho et al*.* (2021) claim that the use of social media and distorted news about COVID-19 are factors that increased stigma [6]. In an effort to diminish the effects of this uncontrolled information (or misinformation), the WHO published a situation report indicating that we were in a context of "infodemia" (overabundance of information of varying accuracy), underlining the need to control false hoaxes and over-information.

There is also a relationship between perceived threat, fear and public stigma. Corrigan et al. (2002) for example, relate the social stigma experienced by people with psychological disorders to fear in the general population [4]. In our case, Cho et al. (2021) point out that COVID-19 was perceived as a threat and when resources to deal with it were limited, it was blamed on minorities, in this case the Asian population [6]. According to some authors, discriminatory behaviour towards the Asian population has been common in different countries around the world, such as the US, France, the UK and Italy [10, 11] as well as in Latin America and Brazil [8]. In addition, anti-Asian xenophobic attitudes have taken different discourses and forms, including derogatory behaviour and rejection of tourists, migrants, residents or even nationals of Asian descent [8].

In addition to the existing stigma, since the onset of the pandemic, people with physical characteristics similar to those of the East Asian population have been subjected to an increasing level of discrimination on a global scale, ranging from verbal abuse to physical assault. Although the Chinese population has been the most affected due to its association with the origin of the pandemic, this discriminatory practice impacts individuals of Japanese or South Korean descent and nearby areas. Because of the erroneous assumption that individuals with East Asian traits are of Chinese nationality, it has led to the invisibility of other ethnic identities [12–14]. This process has even resulted in the terms ‘Asian’ and ‘Chinese’ being used interchangeably, both in racist discourses and in everyday language [14], which has made the stigma behind COVID19 more complex. [14].

In terms of the impact of stigma, discrimination itself is presented as a key risk factor for disease [10]. For example, in the case of migrants, the "healthy migrant effect" is well known, i.e. the improved health conditions of some migrant groups upon arrival in the host country. While this effect is known to fade over time due to acculturation, discrimination has also been shown to play an important role in this deterioration, as it can increase the speed of health deterioration in these populations [15]. In this regard, numerous authors have found a significant deterioration in the physical and psychological health of the Asian migrant population since the emergence of COVID [7, 16, 17]. Particularly, this negative effect of the COVID-19 pandemic on the Asian population has been detected in women [16].

Given the evidence of the existence of public stigma against the Asian population in other parts of the world and its negative impact in terms of public health, it is necessary to study this phenomenon in Spain. The aim of the present study is to explore, to the best of our knowledge for the first time, the public stigma suffered by the East Asian population living in Spain related to COVID-19 and its impact on their perceived health. As secondary objectives, this study aims to examine protective factors and proposed approaches to address public stigma towards the Asian population.

## Method

### Design

This empirical study was carried out in Seville (Andalusia, Spain). The design was cross-sectional and qualitative. According to Leininger (1991), it is essential to consider all those global factors that influence health, such as worldview, language, ethnohistory and the environmental context of individuals [18]. This is why qualitative studies are crucial in the field of health research [19].

### Participants

The sample consisted of 26 people, of whom 17 were women and 9 men. The age of the participants was between 18 and 47 years old. Regarding the place of origin, most of the participants came from South Korea, 16 people; 4 from China, 4 Spanish citizens of Chinese descent and 2 from Japan. The composition of the sample is consistent with data from the 2021 Population and Housing Census (Instituto Nacional de Estadística [INE], 2023), which establishes that East Asians born in China, Japan or South Korea are the most predominant in our country [20].

The criteria for inclusion in the study and the socio-demographic characteristics of the participants are further elaborated in Tables 1 and 2 respectively.

**Table 1:** Inclusion criteria

East Asian origin or ancestry (China, Japan and South Korea)
Be resident in Spain or have been so during the last few months since the start of the COVID-19 pandemic
Age between 18 and 55 years old
Be fully able to answer questions, understand what is being communicated and express themselves correctly in any of the following languages: Korean, English or Spanish
Voluntarily agree to participate in the study after reading the informed consent form and having any questions regarding any part of the research process answered

**Table 2:** Socio-demographic characteristics of the participants

Variables	No. of participants	% of participants
Age Range		
* Between 18 and 34 years old*	18	69%
* Between 35 and 50 years old*	8	31%
Gender		
* Woman*	17	65%
* Man*	9	35%
Educational Level		
* Mid-level Studies*	5	19%
* University studies*	21	81%
Country of Birth		
* South Korea*	16	62%
* China*	4	15%
* Spain*	4	15%
* Japan*	2	8%
Duration of the Stay in Spain		
* Less than 3 years residing in Spain*	7	27%
* Between 3–10 years residing in Spain*	8	31%
* More than 10 years residing in Spain*	11	42%
Reason for migration		
* Migration for studies*	13	50%
* Labour migration (personal or family)*	5	19%
* Lifestyle migration*	3	11%
* Adoption or birth in Spain*	5	19%

### Instruments

A semi-structured interview (Table 3) was conducted in two parts: first, an initial phase for demographic data, followed by 12 open-ended questions focused on exploring experiences of stigma. Due to the characteristics of the sample and being one of the languages accepted in the inclusion criteria, this interview was translated into Korean and reviewed by a member of the community in order not to violate the cultural sensitivity of the participants. In order to facilitate communication with the participants, the script was not strictly followed.

**Table 3:** Interview script

Name:
Age:
Sex:
Country of birth:
Educational level:
Place of Residence:
Duration of stay in Spain:
Current employment situation:
Civil status:
1.- Have you ever experienced (prior to the start of Covid-19) any kind of stigma since you have been living in Spain? From your point of view, you can refer to any kind of attitude that made you feel stigmatised
2.- If the answer is yes, could you tell us some examples?
3.- How did you feel about these situations?
4.- Before living in Spain, had you heard about the stigma attached to the Asian population in the country? What is your perception now?
5.- What do you think is the general opinion that Spaniards have of Asian Migrants?
6.- Have you suffered any kind of stigma since the beginning of the Covid-19 pandemic? Would you relate it to the virus?
7.- If the answer is yes, could you give us some examples? When did these events occur?
8.- Do you consider that this stigma has affected your health in any way (biological, psychological, social and spiritual aspects)?
9.- If the answer is yes, could you please specify in what way?
10.- How have you coped with these situations?
11.- Have you received any kind of external assistance?
12.- Would you propose any type of approach or recommendation to deal with these situations? Please specify
Thank you for your collaboration and attention

The interview content was divided into four parts: pre-COVID-19 discrimination and health impacts (1st-3rd questions), stigma relationship between Spain and Asian immigrants (4th-5th questions), post-COVID-19 discrimination and health impacts (6th-9th questions), coping strategies, help and recommended approaches (10th-12th questions).

### Procedure

**Fig. 1:**
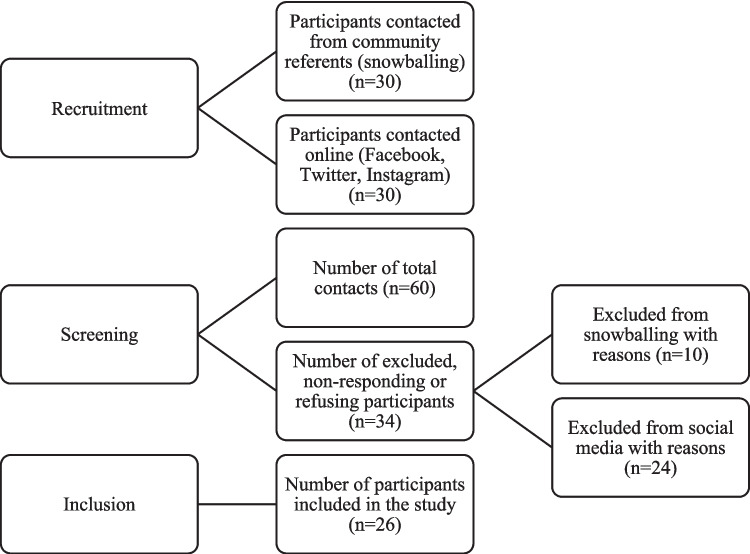
Participant recruitment flowchart

Data was collected between September 2020 and June 2021. Recruitment was carried out by snowballing. This type of technique is quite useful in populations that are difficult to access or contact, such as migrant populations [21]. The first contact was developed through the help of referents in each community and through social media. Subsequently, as illustrated in the graph below, a snowballing procedure was employed until all informants (n = 26) were located (Fig. 1).

Prior to the interview, each participant completed the corresponding informed consent form. The average length of the interviews was 57 min, with a maximum of 70 min and a minimum of 44 min. A voice recorder was used, and the community reference person was present to help with the interpretation.

### Data Analysis

A thematic analysis was chosen [22] whereby a theme or category can be defined as an important statement about the data related to the research questions and involves a meaningful articulation of the participants' responses. The categories found were interpreted and analysed according to the context and experiences of the participants as set out from a phenomenological perspective [23, 24]. The authors analysed all the interviews separately by pooling the categories found in a joint meeting. The interviews were then re-analysed considering new categories. Only those categories and interpretations on which there was agreement between the two authors were included in the subsequent analyses. This process was carried out three times until saturation.

To determine the reliability of the qualitative study, the criteria proposed by Lincoln and Guba (1985) were followed, which include credibility, transferability, dependence or consistency and confirmability [25].

### Ethical Issues

This work respects the anonymity and confidentiality set out in “Ley 3/2018 de 5 de diciembre, de Protección de Datos de Carácter Personal” (Data Protection Law 3/2018, 5th of December) [26] and the ethical principles included in the Declaration of Helsinki [27]. In addition, all participants were informed of the objectives of the study, methodology and implications of their collaboration through both verbal and written informed consent, and approval was obtained from the Biomedical Research Ethics Portal (University of Seville, No: 0339-N-17).

## Results

**Fig. 2:**
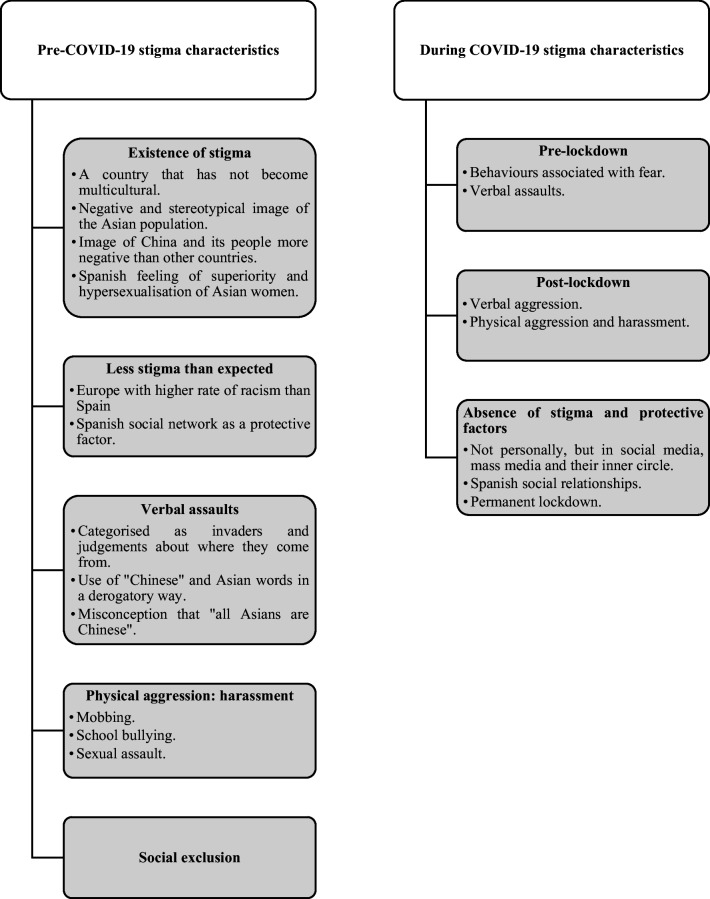
Category map: classification of stigma

**Fig. 3:**
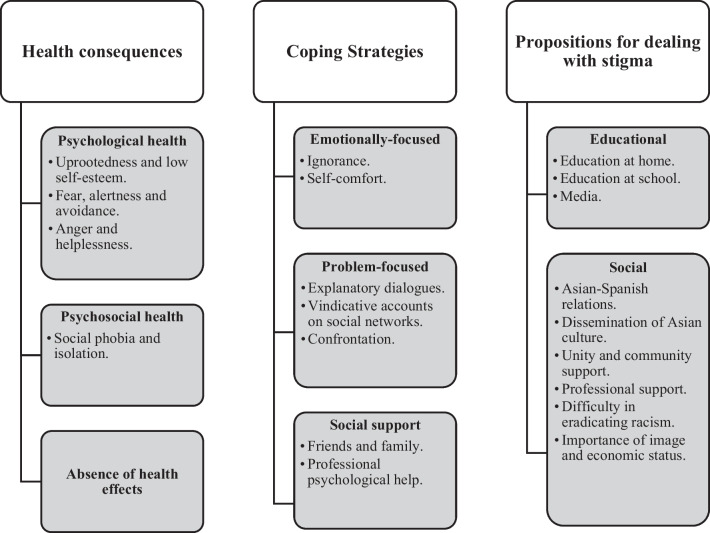
Map of categories: health consequences, coping strategies, social support and propositions for dealing with stigma

The initial categories correspond to the questions posed in the interview script. Subsequently, subcategories were established as a result of the analysis of the answers received by the participants in the study. These in turn are ordered by frequency. The outcome of the analyses is presented in two category maps, one explaining the stigma suffered (Fig. 2) and the other reflecting the consequences on health, coping strategies, social support and proposals for addressing it (Fig. 3).

To facilitate the understanding of this section, it is important to clarify that the term "stigma" was understood by the participants as "discrimination", one of the components of stigma, or “racism”, a type of stigma associated with race. Although the words originally used by the participants in the excerpts will be respected, the concept of stigma will be used in most of the participants' explanations. The following section provides a brief overview of the categories, with selected excerpts used to illustrate the points.

### Pre-COVID-19 Stigma Characteristics (Fig. 2)

In the case of people who arrived in Spain after lockdown, this question will not be subjected to analysis. Specifically, these were interviews E2, E7, E25.

#### Existence of Stigma

Spain was categorised by some participants and those close to them as "one of the worst countries to travel to because of racism towards Asians" (E7, personal communication, 16 December 2020) or as "a country that has not become multicultural" (E11, personal communication, 21 December 2020) and with more "discrimination" today. Similarly, those who were born in Spain, arriving in the country by adoption or at a very young age, confirmed the existence of stigma today.

*Female, Korean. "(...) they make the slant-eyed gesture or tease us by calling us Chinese and run away or hit us. I have experienced racism once".* (E25, personal communication, 02 June 2021).

The participants also highlighted the negative and stereotypical image of the Asian population, who are considered dirty, degradable, people who develop illegal businesses, and even distrustful and isolated beings with no capability to integrate into society. They were also labelled as invaders, people who come to take away work or even a source of ridicule (jokes, they eat all kinds of living beings). When interacting with Spanish people, they were surprised to see a friendly and pleasant personality.

*Female, Spanish of Chinese descent. "(...) that many are not able to integrate well into Spanish society and that they have problems communicating well in Spanish. Also, there are the typical stereotypes regarding Chinese people eating all kinds of living things, including pets".* (E20, personal communication, 03 April 2021).

Some participants were of the opinion that the image of China and its people was worse than that of other Asian countries.

*Female, Korean. "Then all of a sudden she started insulting Chinese people (...) my academy teacher, she has a good image of Koreans and Japanese, but she doesn't seem to like Chinese people very much".* (E6, personal communication, 15 November 2020).

There was a perceived feeling of superiority in Spaniards, who considered Asian people inferior. This is coupled with the thought that people "who are small, thin" are more susceptible to discrimination. Within this point, participants highlighted the hypersexualisation of Asian women.

*Female, Spanish of Chinese descent. "Women are seen as submissive and weak (...) hypersexualised, being the object of "morbidity" by white heterosexual men, who feel superior".* (E20, personal communication, 03 April 2021).

#### Less Stigma than Expected

In contrast to the previous category, participants were interviewed who, after their stay in Spain, considered that there is less racism than they expected. They even shared the idea of a Europe with a higher rate of racism, highlighting its presence in countries such as France, England and Italy.

*Female, Korean. "(...) Generally I was told that there is a lot of racism in Europe. Not long ago in France there was too, (...)I just knew that wherever you go there is Asian racism".* (E3, personal communication, 3 October 2020).

Some of the participants referred to their social network, e.g. Spanish friends, or partner, to explain why they did not perceive a high level of stigma. Other participants, as seen in the excerpt below, explained that stigma towards the Asian population is not as negative.

*Female, Spanish of Chinese descent. "It is not as negative as it is towards the rest of the ethnic groups due to economic issues, I think, but there is still rejection and mockery".* (E15, personal communication, 17 February 2021).

#### Verbal Assaults

Most interviewees reported that the stigma they suffered was mainly verbal. They were categorised as invaders, their origin was criticised and they even reported hearing expressions such as "go back to your country/home".

*Female, Spanish of Chinese descent. "I have been called "Chinese cunt" to my face thousands of times in high school when I was 13 years old. I've been shouted "go back to your country" I don't know how many times, and not only young people, older people too.* (E14, personal communication, 8 February 2021).

It is worth noting that the use of the term "Chinese" or words derived from Asian languages, which were perceived by the intonation as "insulting", "racist" or "discriminatory".

*Male, Korean. "I don't feel bad when they call me "Chinese" or "nihao", but I understand from their tone of voice that they do it to annoy me...".* (E8, personal communication, 16 December 2020).

Following this line, most of the participants agreed that it is common for Spaniards to think that all Asians are Chinese because of their physical characteristics. The process of Asian homogenisation for non-Chinese people was perceived as an uncomfortable experience due to the phenomenon of invisibilisation.

#### Physical Aggression: Harassment

Some participants described experiences of bullying, highlighting bullying at school (E10, E11), mobbing or even sexual assault.

*Female, Korean. "Some elderly men would come up to me for no reason and kiss me on the cheek and tell me to go home with them...".* (E9, personal communication, 18 December 2020).

#### Social Exclusion

In this case, it is found discriminatory behaviour marked by exclusion.

*Female, Korean. "people in Barcelona don't have much interest in other people (...) I felt like I was excluded, you know? More excluded than normal".* (E3, personal communication, 3 October 2020).

### During COVID-19 Stigma Characteristics: Pre-lockdown

#### Behaviours Associated with Fear

First, there were behaviours associated with fear, such as covering the nose and mouth. In addition, there was avoidance, "not associating with Asian people", refusal to rent a house, and derogatory looks. At the beginning of the pandemic, people thought that the virus came from China, and as there were not many cases in Europe, people began to question and stigmatise Asians. This was experienced by adults as well as children and spread geographically, including on buses, in parks, neighbourhoods, restaurants and workplaces. Such behaviour was linked by some participants to inaccurate media reporting.

*Female, Chinese. "My neighbour covered herself whenever she saw me in the corridors, but not with other people. Also the neighbours in my village said it was better not to hang out with people with Asian features".* (E10, personal communication, 27 December 2020).

#### Verbal Assaults

Young people tended to be verbally violent. In addition, a type of verbal aggression appeared that had not been mentioned before, namely calling the person "COVID".

*Female, Korean. "I heard a nasty voice saying: ‘look they are Chinese’. There are strange people who think all Asians are the source of the virus. In March a boy who looked like a student kept shouting at me that I was Chinese and to go away".* (E9, personal communication, 18 December 2020).

*Female, Spanish of Chinese descent. "Mocking and offensive comments about covid. A group of teenagers that I came across in the street started shouting ‘coronavirus´ at me and making weird sounds as if they were speaking Chinese. Without knowing me at all and simply because over the mask you can tell I have Asian features".* (E20, personal communication, 03 April 2021).

### During COVID-19 Stigma Characteristics: Post-lockdown

After lockdown, the typology of the stigma suffered was accentuated in all the mentioned spheres and acquired new nuances as can be seen below.

#### Verbal Assaults

The most common verbal assault was the naming of the Asian person as "COVID". In this way, the reification and stigma suffered could be seen, turning the person into a disease which today is considered a public health hazard.

*Female, Korean. "Every day at least once I have experienced discrimination. I have always heard them call me Chinese, coronavirus and tell me to go to China... things like that... One day when I got on the bus, they insulted me by calling me coronavirus to my face".* (E7, personal communication, 16 December 2020).

As before the pandemic, homogenisation of the Asian population continued to be present as a stigmatising behaviour.

*Female, Korean. “On the street, teenagers suddenly came up to me and said: ‘Hey Chinese, why did you go out? Be careful with your bag, we're going to steal it. Go to China!’ That's how they threatened me”.* (E7, personal communication, 16 December 2020).

#### Physical Aggressions and Harassment

Physical aggressions were accentuated after lockdown, one of them suffered by the participant herself or by people close to her.

*Male, Korean. "One of my Korean friends here in Valencia has suffered a lot. A few days ago, he was walking down the street and a Spanish guy started calling him 'Chinese, virus' and punched him in the face. He started bleeding”.* (E8, personal communication, 16 December 2020).

Harassment, of a discriminatory and sexual nature, also recurred.

*Female, Korean. "This December an elder man tried to kiss me again and it was very unpleasant".* (E9, personal communication, 18 December 2020).

*Female, Korean. "Suddenly someone started banging on the window. Then more kids came and started banging on our window like they were crazy, and we just ignored it”.* (E2, personal communication, 12 September 2020).

### During COVID-19 Stigma Characteristics: Absence of Stigma and Protective Factors

Contrary to the previous ones, some participants indicated that they had not been the direct object of the stigma themselves. However, they may have been indirectly exposed to it through confessions of individuals with whom they were closely acquainted or through social networks.

*Female, Spanish of Chinese descent. "Apart from the incident I mentioned before, I have not suffered any other type of discrimination directly against me. Although I have heard of cases from my inner circle”.* (E20, personal communication, 03 April 2021).

A relationship was observed between the absence of stigma in some participants and a long-term stay in Spain, linked to established social relationships with Spaniards; to the good management of the pandemic in South Korea and even to the context of the world of flamenco as a protective factor. In some extracts from participants who have not experienced stigma, a certain stigmatising paternalism could be detected when referring to Chinese people as *chinitos* (little Chinese) or only mentioning it in this population.

*Male, Japanese. "I haven't suffered from it. I suppose it's because I've been living here for many years, I'm like one of them. In the world of flamenco this doesn't usually happen. Although I know of some ‘chinitos’, friends of mine, who have suffered from it..."* (E22, personal communication, 07 April 2021).

In addition, on one occasion there was an absence of stigma which is justified by the participant as being in permanent lockdown, which hindered social relations and thus the existence of discrimination.

*Female, Chinese. "No, I've been confined all the time."* (E11, personal communication, 9 January 2021).

### Health Consequences of Stigma (Fig. 3)

A distinction was made according to the dimensions that make up health, following the statements of the interviewees.

#### Psychological Health

The feeling of non-belonging and low self-esteem stood out. In particular, it is interesting to mention that even among people who had been born or raised in Spain, this feeling existed A distinction is made between "normal" Spaniards and themselves, categorised as "weird" or "not real Spaniards".

*Female, Korean. "When I would meet my friends and say hello to other people, they would ask them why they were with us. I felt like we were bothering them all of a sudden. At that time I felt a bit sad. Am I the only one who will spread the virus? (...) That's why I always thought that no matter what, I will always be a foreigner to them."* (E3, personal communication, 3 October 2020).

In line with "being permanent foreigners", there was a constant worry of being discriminated against in any place or situation. Accompanied by a behaviour of fear, alertness and avoidance, or even the thought that those who previously treated them well might discriminate against them.

*Male, Korean. "Before going through a group of people, I was already worried that they were going to say something to me. And also after COVID, like going into a restaurant, I could expect that they were going to do something to me. A normal person shouldn't have to worry like that".* (E1, personal communication, 6 September 2020).

Feelings of annoyance were also found, sometimes accompanied by responses that clarified where the person was coming from. In conjunction with reactions of anger, helplessness or frustration.

*Female, Korean. "On a psychological level, I started to doubt why I have to spend my money to learn the language and culture of a country that does not respect me...".* (E2, personal communication, 12 September 2020).

#### Psychosocial Health

In some cases, social phobia or isolation was mentioned. It is necessary to understand that the social aspect is intrinsically linked to the psychological aspect, as mentioned by several participants.

*Female, Korean. "Psychologically and socially. I'm afraid to go to a bar or restaurant, sometimes I developed some kind of social phobia. I couldn't look into people's eyes because maybe they were going to hit me in the head again or call me Chinese".* (E7, personal communication, 16 December 2020).

#### No Health Effects

Three of the participants did not perceive any effect on their own health.

*Male, Korean. "I have ignored everything I have been told and I have gone on with my life”.* (E8, personal communication, 16 December 2020).

### Coping with Situations of Discrimination

#### Emotionally-Focused

Avoidance was the predominant coping strategy. For example:

*Female, Spanish of Chinese descent. "Breathing and ignoring".* (E15, personal communication, 17 February 2021).

Two of the participants mentioned self-comfort, convincing themselves that the world is full of good and bad people, comforting themselves.

#### Problem-Focused

Explanatory dialogue as a means of clarifying the situation and attempting to resolve the conflict was presented as a key coping measure.

*Female, Korean. "Despite all the difficulties to cope with, I always try to go head-on, and if someone attacks me in any way, I defend myself and tell them that despite everything, please, have some respect”.* (E19, personal communication, 30 March 2021).

One of the interviewees mentioned the development of a social media account that has allowed her to connect with the community and defend her rights.

*Female, Chinese. "Creating a social media account to vindicate our struggle and rights. @antirracismoasiatico".* (E10, personal communication, 27 December 2020).

On the other hand, confrontation was a strategy used by fewer participants. In these cases, through direct verbal confrontation which sometimes led to acts of fighting.

*Female, Korean. "I have shouted at them telling them to leave me alone or asked them very angrily what they had said to me".* (E9, personal communication, 18 December 2020).

#### Social Support

More than 2/3 of the participants reported not having received help at any of the times when they had been victims of anti-Asian stigma. However, among those who did receive help, there was an absence of stigma in the presence of Spanish friends, who were responsible for responding to the aggressors.

*Female, Spanish of Chinese descent. "Some friends of mine have jumped in...".* (E12, personal communication, 10 January 2021).

One participant noted the importance of family support from childhood in understanding and dealing with stigma.

*Female, Chinese. "My mother used to ask me about this and made me understand the incongruity of discriminating basing on one’s country of origin, although I didn't understand it until I grew up”.* (E18, personal communication, 16 March 2021).

Some participants highlighted the importance of professional assistance, particularly psychological support, in coping with adverse experiences of stigma. The sole individual who sought psychological assistance was among those who had been transnationally adopted. Furthermore, the remaining participants in this group perceived this to be a valuable and beneficial avenue of support.

### Propositions for Dealing with Discrimination and Stigma Situations

#### Educational

Education was presented as the main approach. Starting at home, as stigma has increased among young people. At the same time, there was a call for more content in schools and in the media on issues such as racism, discrimination and cultural diversity. Along these lines, the mention of human rights education is interesting. Research and scientific dissemination were also suggested.

*Male, Japanese. "The eradication of racism, xenophobia or sinophobia needs a lot of research. It is part of the sustainable development goals. I think this research can help".* (E22, personal communication, 07 April 2021).

#### Social

There was a shared opinion that the relationship between Asians and Spaniards was essential, favouring understanding between the two. In addition to sharing Asian culture through media such as food, music and customs in order to promote a better comprehension of it.

*Female, Korean. "I think, if we teach Asian culture to the public, for example Korea through food, music, customs... I think they get an appropriate image of our culture”.* (E3, personal communication, 3 October 2020).

The importance of unity, community support and psychological help is mentioned, highlighting the need for professional support to accompany the affected person and the involvement of witnesses of the aggressions.

*Female, Spanish of Chinese descent. "Psychological help to help understand that it is not something we can control and that it should not affect us, we have the same rights and we don't have to prove anything to anyone".* (E12, personal communication, 10 January 2021).

Two of the participants acknowledged the current difficulty of eliminating racism in society. They explained that it is not only exclusive to Spain and the Asian population, but that it is widespread throughout the world. It is seen as inevitable, utopian or difficult to change on a small scale.

*Male, Korean. "I don't think racism is going to disappear easily. Because it exists not only in Spain, but in any country. (...) I think the end of racism is a bit utopian or ambitious. So, racism by society is inevitable".* (E5, personal communication, 19 October 2020).

In addition, they suggested the importance of a good image as a protective factor, relating it to high economic status*.* This established a link with possible cultural assimilation.

*Female, Korean. "If we show a nice, clean image... they will think: the Koreans, the Chinese or the Japanese that I know are very good. So to solve racism, you both have to make an effort together”.* (E6, personal communication, 15 November 2020).

## Discussion

To the best of our knowledge, this study is the first to explore the public stigma related to COVID-19 experienced by the East Asian population living in Spain and the subjective perception of its impact on health. Overall, there is general agreement with the previously consulted literature, however, this study also presents some innovative findings.

The majority of participants in this study said that there has been an increase in public stigma following the pandemic. Furthermore, two-thirds of them said that it existed previously and that they had already been victims of it. This was most strongly affirmed by Spanish participants of Asian descent, Chinese-born people who have been adopted and younger migrants. Empirical evidence shows that young people and second-generation migrants are more sensitive to discrimination and stigma in Europe [11, 28]. However, older migrants report that they did not experience discrimination prior to the pandemic. Previous studies indicate that older migrants may deny the racism and discrimination they experienced [11]. In addition, the superiority of stigma in countries such as the UK and France compared to Spain according to participants is also confirmed, which is consistent with previous research [10, 11].

Regarding the perception of stigma, most of the interviewees underlined that in Spain there is a widespread misconception that Asians are Japanese or Chinese. To this fact was added the stigma caused by the outbreak of the COVID 19 pandemic [16]. The research revealed the presence of negative stereotypes, including the portrayal of these individuals as unclean and unpleasant, and as people who consume unusual animals. Asian food culture has been categorised as primitive or dirty since ancient times in Western countries [11]. This belief emerged again due to COVID-19, as images of the Wuhan market where wild animals were sold as food were shown, increasing the stigma. On top of that, videos of bat soup were disseminated on social media, pointing to Chinese people as targets and culprits of COVID-19 [10, 29]. Interestingly, we found a paradox between a certain good image associated with Asians and the perceived feeling of superiority in Spaniards. This has long been detected in the US, where Asians are categorised as the 'model minority', with a good image as hard-working, intelligent and represented by a good economic and educational level. But at the same time, they are treated as "perpetual aliens", as they are never considered worthy of US status [1].

In terms of typical discriminatory behaviour, it has been observed that the Asian population is frequently held responsible for the spread of the virus and referred to as "COVID." This phenomenon has been designated "COVID-19 personification" [30]. This is replicated in other countries, such as Chile [29] or India [9]. On the contrary, Koller et al. (2021), working with a German population, found that only a small proportion of respondents showed stigmatising behaviour [31]. According to these authors, this good result is associated with the age of the participants, whose average age was 33.37 years. [31]. It is interesting to note that several of the interviewees in this study mentioned that their aggressors were teenagers, although after lockdown these attitudes were also widespread among adults and older people. In this sense, it is worth asking about the influence of age on the existence of stigma towards this population.

Pandemic-related assaults, fear-related behaviours and media stigma have been reported by participants in this research [32] Fear is mentioned by several authors as a risk factor for discrimination [3, 6]. According to the participants, the fear-associated behaviours started due to an excess of alarming information in the media, in which COVID-19 started to be called the "Chinese virus" [1, 8, 9, 33]. In order to understand the phenomenon of stigma towards the Asian population, it is important to take into account the collectivist bias of their cultural heritage. From the moment that the use of masks became mandatory at the beginning of the pandemic in countries such as China, Japan or Korea, this was passed on among friends and relatives around the world to encourage collective care of the population. Thus, while a collectivist Asian culture considered not wearing a mask to be stigmatising for a lack of civic awareness and citizenship, Western individualistic societies rejected this stance and considered it a sign of illness, criminality or individual weakness [34]. Thus, participants allude that they suffered social rejection for the use of facemasks at the beginning of the pandemic [8, 10, 11] by the Spanish population in public places, transport or even when looking for housing. Once the pandemic shook Europe and after lockdown in Spain, these behaviours evolved in such a way that the rejection of the use of masks turned into fear of contagion. From that moment on, the interviewees experienced the rejection of many citizens when they noticed that they put on their masks only when someone with Asian features approached them. In addition, social media apps became transmitters of stigma directly and indirectly [35] and those who barely perceived stigma on the streets were able to experience it through these media.

On the other hand, respondents who had not experienced public stigma justified it with a permanent situation of lockdown, a primarily Spanish social and support network, and even a protective work context. These situations are in line with research by Lee & Waters (2020) where the support network, the racially diverse community and the deprivation of social relations due to lockdown were presented as protective and buffering factors for discrimination [17].

Regarding the consequences on health, it can be observed that they are of a diverse nature. In the area of mental health, the feeling of being perpetual foreigners, the feeling of not belonging to the community and the low self-esteem caused by this are particularly relevant. These feelings can be associated with the label of "strangers in their own land" [17]. The problem is that the pandemic has fortified this sense of being a foreigner [1, 33, 36] causing stress disorders that can lead to a variety of diseases [1, 33, 36]. Thus, two-thirds of participants experience feelings of distress, frustration, anger and helplessness.

It is possible to find a strong connection between the social and psychological consequences of stigma. Some participants reported that they had developed a fear of social interaction as a result of their distress and fear of being discriminated against. Despite their efforts to gain Spanish language proficiency, some individuals had not yet reached a level of proficiency that would enable meaningful communication. The context of a pandemic further complicates the process of establishing robust language connections, given the inherent communication difficulties and absence of pre-existing networks that are characteristic of a situation of prolonged social confinement [33, 37]. In turn, social isolation can cause a decrease in income, problems with employment and a decrease in social networks [38]. Beyond the health issues discussed above, there is widespread concern in the literature that COVID-19-related anti-Asian stigma may lead to increased racial violence and the perpetuation of racism towards these communities [16, 33].

Coping strategies employed by participants were diverse, with ignorance standing out. According to Yashadhana et al*.* (2021), one of the coping strategies used by racial minorities in situations of discrimination is remain silent about racism by "silently enduring the pain" which may contribute to isolation and perpetuate stigma. Confrontation was also present [39]. Brondolo et al. (2009) point to confrontation as a buffer for depressive symptoms resulting from discrimination in Korean migrants [40]. On the other hand, the development of social networks had also turned them into an effective coping strategy [11, 41]. Following this line, support networks are presented as the main source of comfort, and particularly the Spanish network as a protective element of discrimination. This reinforces the importance of social support as a coping mechanism and a protective factor against discrimination [37].

As proposals for approaching the issue, it is essential to educate in equal rights for all collectives [34]. Equally, it is important that this education takes place not only in schools and homes, but also in the media, which serve as a source of global information [42]. Nevertheless, some participants are sceptical about the eradication of discrimination, proposing assimilation as a solution. However, in our opinion, this would not be an adequate prescription due to its role as an aggravating factor in migration stress [43] and its ability to counteract the healthy migrant effect [15]. In this way, the health of migrants would be damaged by assimilating into the Spanish culture. An example of this would be the increase in smoking among Chinese women or the approach to a fatty nutritional pattern that could lead to cardiovascular diseases among Chinese migrants living in southern Spain [44].

### Strengths, Limitations and Future Research

This study has several limitations. It should be noted that the recruitment of participants took place during a pandemic period, which made access and participation difficult. It would be necessary to increase the number of participants from Chinese and especially Japanese backgrounds. The vast majority of the sample has a university education. It is to be expected that a lower level of education and thus participation in different social contexts would lead to significantly different experiences of stigmatisation. Therefore, it would be appropriate to explore stigma in less educated people. One would also expect a social desirability bias to be implicit in the responses, where one avoids being seen as a "victim" [45]. This, however, would imply that the stigma detected would be less than the actual stigma. The cross-sectional nature of the study only shows us the information collected during the time of recruitment of participants. It would be interesting to conduct a longitudinal study to see if COVID-19 continues to influence discrimination against the Asian population, in addition to an in-depth exploration of the health of these groups.

Despite the limitations described above, the research has several strengths, as the findings provide the basis for the development of future research and intervention proposals. After the return to ‘normalcy’, it would be necessary to explore whether the level of stigma towards the Asian population has returned to pre-pandemic levels or is still higher. It is essential to identify the needs of these groups, develop preventive and educational programmes and promote the union of Asian communities, favouring their empowerment. At the same time, it is also important to have culturally competent professionals at all levels of care, who are able to recognise and prevent discrimination and provide responses that are adapted to the health problems of Asian communities. Understanding the stereotypes and cognitive distortions that underlie the stigmatisation of specific groups is essential for designing targeted interventions. This study contributes to the understanding of stigma towards East Asians in Spain and thus to the effective design of such interventions.

## Data Availability

Upon request.
